# Students choosing fat-free chocolate milk during school lunch consume more calories, total sugar, protein, minerals and vitamins at lunch

**DOI:** 10.1017/S1368980021000161

**Published:** 2021-05

**Authors:** Janet G Peckham, Jaclyn D Kropp, Thomas A Mroz, Vivian Haley-Zitlin, Ellen Granberg

**Affiliations:** 1 US Food and Drug Administration, Office of the Commissioner, 5001 Campus Drive HFS-020, College Park, MD 20740, USA; 2 Food and Resource Economics Department, University of Florida, Gainesville, FL, USA; 3 Andrew Young School of Policy Studies and the Federal Reserve Bank of Atlanta, Department of Economics, Georgia State University, Atlanta, GA, USA; 4 Food, Nutrition, and Packaging Sciences Department, Clemson University, Clemson, SC, USA; 5 Department of Sociology and Anthropology, Rochester Institute of Technology, Rochester, NY, USA

**Keywords:** NSLP, School lunch, Flavoured milk, Plate waste, Selection and consumption

## Abstract

**Objective:**

To examine how milk consumption varies by milk choice and measure the association of milk choice on the nutritional and energetic content of National School Lunch Program (NSLP) lunches.

**Design:**

An observational plate waste study using the Digital Photography of Foods Method.

**Setting:**

Data were collected from two suburban South Carolina schools in one district during February and March 2013.

**Participants:**

Totally, 968 NSLP lunches selected by 619 kindergarten to fifth grade students.

**Results:**

Most students chose chocolate milk (75 %). A multinomial logit model indicated milk choice varied significantly by sociodemographic characteristics. An ordinary least square regression indicated that consumption rates of low-fat white milk were 8·5 % lower than fat-free chocolate milk (*P* = 0·039) and milk consumption rates varied statistically by sociodemographic characteristics. Ordinary least square regressions found that the consumption of energies and nutrients from NSLP lunches varied with sociodemographic characteristics and milk choice; students selecting chocolate milk consumed 58 more energies (*P* < 0·001) and 10 more grams of total sugar (*P* < 0·001) than students selecting low-fat white milk from their NSLP lunches. Students consumed statistically similar energies and nutrients from the non-milk components of their meals.

**Conclusions:**

Students selecting chocolate milk consumed more energies and nutrients from their NSLP lunches with the increases in consumption attributed to the milk component of the meal. The findings have implications for recent changes to NSLP guidelines that allow schools to offer both low-fat and fat-free flavoured milk, reversing the previous ban on low-fat flavoured milk under the Healthy, Hunger-Free Kids Act.

Milk is an important source of high-quality protein, Ca, vitamin A and vitamin D, and milk has as a long history of inclusion in federally funded school meal programmes. Even prior to the introduction of the National School Lunch Program (NSLP) in 1946, schools were serving milk under federal assistance programmes^(1)^. There is a long-standing debate surrounding serving flavoured milks in schools and its impact on child nutrition. Flavoured milks offer similar nutrient profiles relative to unflavoured milk but include added sugars and flavourings, such as chocolate or strawberry, to make the milk more enticing. Proponents of offering flavoured milk as part of school meal programmes argue flavoured milk provide a nutrient-dense beverage with less added sugar than other sugar-sweetened beverages. But those opposed argue added sugars contribute to the obesity epidemic and the nutritional benefits of flavoured milk do not outweigh the health risks associated with childhood obesity^(2)^.

In an effort to balance flavour with fat and energy content, in the fall of 2012, as a component of the Healthy, Hunger-Free Kids Act (HHFKA) of 2010, districts and schools could no longer offer low-fat flavoured milks as part of federally funded school meal programmes; flavoured milks could only be offered if they were fat-free. Some school districts, including prominent cities such as Washington, DC and San Francisco, chose to ban flavoured milks altogether^(3,4)^. In October 2017, citing the health benefits of milk, the historical inclusion of milk in school-based federal meal programmes, declining milk consumption in schools, declining participation in the NSLP and the importance of promoting milk consumption to ensure the health of school-age children, the School Milk Nutrition Act of 2017 was introduced into the House of Representatives (H.R. 4101). It proposed amending the National School Lunch Act to allow schools and districts flexibility to determine which milkfat choices to offer. In December 2018, the US Department of Agriculture revised the HHFKA to allow schools and school districts the option of offering flavoured, low-fat milks as part of the NSLP in addition to flavoured, fat-free milk^(5)^. Some school districts around the country continue to ban flavoured milks, others such as New York City are considering eliminating flavoured milks^(6)^, while other districts, such as New Haven, Connecticut, are placing flavoured milks back on the menu^(7)^.

Clearly, serving flavoured milks in schools remains a topic of much national debate with the School Milk Nutrition Act calling for ongoing research of milk consumption patterns in children. Specifically, the call for research indicates the need for a better understanding of the effects of offering flavoured milks on the consumption of energies, fats and sugars. This study answers that call by examining the relationship between NSLP participants’ milk choice and the nutritional content of their NSLP lunches. Since the prevalence of nutrition-related health outcomes such as obesity is highest amongst non-White minorities and socio-economically disadvantaged populations, we examine how this relationship varies across subpopulations of NSLP participants^(8)^. Controlling for the choice of milk and the sociodemographic characteristics of the NSLP participants, we analyse the selection and consumption of energies and nutrients by NSLP participants using plate waste data.

While several prior studies analyse milk consumption and the energy and nutrient intake of milk consumed as part of a NSLP meal^(9,10)^, we analyse the complete meal as well as the milk and non-milk components separately to more clearly understand the role of milk in providing nutrients as part of a NSLP lunch. Furthermore, by linking the plate waste data to student-level data on eligibility to receive free-/reduced-price lunches and demographic characteristics, we are able to examine how choices and consumption patterns vary across gender, race/ethnicity, socio-economic status and grade level. This improves upon prior studies which use aggregate school-level data or do not control simultaneously for these various sociodemographic characteristics^(9–11)^. We hypothesise that students who select chocolate milk consume more total sugar and energies than students who select white milk from the milk component of the lunch. Our unique analysis allows us to go one step further and examine whether students who select chocolate milk consume more energies from other meal components as well or if they trade off the additional energies in chocolate milk by consuming fewer energies from the other meal components. We also examine milk selection and consumption of saturated fat, protein, Ca, Mg, vitamin A and vitamin D as a function of milk choice and sociodemographic characteristics.

## Methods

We measured the nutritional value of school lunches selected and consumed by NSLP participants at two suburban South Carolina elementary schools following the implementation of the nutritional guidelines set forth in the HHFKA of 2010. Each day, the study schools offered three entrées (combined meat/meat alternatives and grain meal components), two fruit, two vegetable and two milk (low-fat white or fat-free chocolate milk) options. In accordance with the HHFKA, students were required to select at least three of the five offered meal components (meat/meat alternative, grain, fruit, vegetable and milk). Thus, students selected and consumed lunches with different nutritional contents. We collected digital images of the children’s lunch selections and their plate waste. From these images, we calculated the consumption of each food item and linked the data to nutritional information. We then linked these data to student-level sociodemographic characteristics provided by the school district to investigate how students’ choice of milk relates to the overall nutritional content of the NSLP meals selected and consumed by NSLP participants.

The plate waste data were collected over a 2-week period in February and March 2013 by a team of data collectors consisting of 3 of the co-authors, 2 graduate student research assistants and 12 other hired graduate and undergraduate students. All members of the data collection team received 8 h of training on the Digital Photography of Foods Method by Pennington Biomedical Research Center (PBRC) researchers prior to the start of data collection^(12–14)^. Data were collected for five consecutive days at each of the two elementary schools, one school each week. The data collection team used digital video cameras to capture images of each student’s food and milk selections before eating and plate waste after eating. Obstructions such as utensils and napkins were removed before capturing the images of the trays. Prior to capturing the returned tray image, unconsumed milk was carefully poured from its carton into a clear cup and if all of the milk had been consumed the milk carton was turned on its side. Images of the students’ trays before and after consumption were matched using each students’ unique participant identification number, which were written on clothespins and carefully positioned on the trays to display the identification number before each image was captured. Data were collected for all students selecting a NSLP meal, a total of 6006 paired images. However, to reduce the cost of analysing the images, we chose a random subsample of 1000 paired images stratified on free-/reduced-price lunch status to ensure representative coverage for analysis^(15)^. After removing observations with missing demographic data and one student who chose both fat-free chocolate and low-fat white milk, there were 968 usable observations.

Prior to recording student trays, the data collectors weighed three to five servings of each menu offering. The average weight of each item was used as the standard serving reference weight. Images of each of the weighed items were recorded to be used as visual references during the analysis of the images.

The selected images were analysed by two trained nutritionists at PBRC using a reliable and validated method for measuring a student’s food intake and plate waste as a percentage of a standard serving^(14,16)^. The energies, macronutrients and key micronutrients were calculated for each food item, including milk, using recipe information, production and procurement records provided by the school, and the USDA Food Composition Database^(17)^. Combining the energy and nutrient data with the PBRC’s analysis of the video images, we constructed the total nutrients selected and consumed for each meal and then also separated these into nutrients consumed from the milk and non-milk meal components. We focus on energies, total sugar, saturated fat, protein, Ca, Mg, vitamin A and vitamin D consumed; additional macro- and micro-nutrients were measured but are not reported here.

Each student had 30 min to select their foods, sit down and eat lunch. Students completed purchase transactions using their unique personal identification number, which was linked to account information regarding lunch-price status (free price, reduced price or full price). By matching these personal identification numbers with students’ participant identification numbers, we were able to match the tray images to sociodemographic information on students’ race/ethnicity, gender, and grade level and eligibility to receive a free- or reduced-price lunch (lunch-price status) obtained from school administrators. The race/ethnicity and gender data were self-reported to the school district by the student’s parent/guardian when the student enrolled in school.

This study was part of a larger study pertaining to the impacts of the HHFKA. Because no identifiable images of the children were recorded, there was a waiver of consent for the photographic study. However, parents were given the opportunity to opt out; 9 % of parents chose to have their child opt out from meal photography. All study procedures were approved by the Institutional Review Board of Clemson University (IRB Protocol 2012–364). Neither children nor parents received compensation for participating in the study.

### Statistical analysis

We used means and standard deviations to describe the data. Specifically, we calculated the average energies and nutrient content of the meals selected and consumed by the student’s choice of milk (low-fat white, fat-free chocolate or no milk). Differences across groups of milk consumers are tested using *t* tests. In addition, we provide the median and the interquartile range for energies and each nutrient content in Appendix A.

We then estimated the likelihood of NSLP participants selecting among fat-free chocolate milk, low-fat white milk or no milk using a multinomial logit model to determine the association between sociodemographic characteristics and student’s milk choice. For each sociodemographic characteristic, we report the estimated change in the likelihood of choosing either fat-free chocolate milk or no milk compared to low-fat white milk, holding the other sociodemographic characteristics constant. To simplify interpretations, we also report how these characteristics impact the no milk choice *v*. the fat-free chocolate milk choice.

An ordinary least squares linear regression was then used to determine how the proportion of milk consumed varied with milk choice, while controlling for sociodemographic characteristics. For this analysis, we limited the sample to the 890 meals containing a milk. To determine the relationship between milk choice and consumption of milk, we included an indicator variable that took the value of 1 if the student selected fat-free chocolate milk and 0 otherwise. Low-fat white milk was the base category and thus the coefficients on the indicator variables represent the difference in the proportion of the milk consumed by students who selected fat-free chocolate milk relative to students who selected the low-fat white milk after controlling for sociodemographic characteristics.

We also used ordinary least square regression analyses to evaluate how milk choice correlates with total energies and nutrients consumed, how milk choice is associated with the consumption of nutrients and energies from the non-milk components of the NSLP meal, and how milk choice is related to the consumption of nutrients and energies from the milk component. Specifically, we ran three regression for energies and each nutrient of interest (total sugar, saturated fat, protein, Ca, Mg, vitamin A and vitamin D). The dependent variable of the first regression was the total consumption of the nutrient (or energies) from the participant’s complete NSLP meal. The subsequent two regressions separated the participant’s consumption into consumption from the non-milk components of the NSLP meal and consumption from the milk component of the NSLP meal; thus, the dependent variable of the second regression in the set was the participant’s consumption of the nutrient (or energies) from the participant’s non-milk lunch components, while the dependent variable of the third regression was the participant’s consumption of the nutrient (or energies) from the milk component of the lunch. To determine the relationship between milk choice and consumption, two indicator variables were included as covariates in these regressions. The first indicator variable took the value of 1 if the student selected low-fat white milk and 0 otherwise. The second indicator variable took the value of 1 if the student selected no milk and 0 otherwise. Fat-free chocolate milk was the base category and thus the coefficient on each of the two indicator variables represents the difference in consumption of the nutrient of interest (or energies) relative to students choosing fat-free chocolate milk. Sociodemographic characteristics are also included as covariates.

In the regression analyses, we include a complete set of day of the week dummy variables and an indicator variable for the school as well as the interaction between the day of week dummy variables and the school variable to account for variation across days and schools. These also control for menu offerings which varied each day. To account for students whose consumptions were measured on more than 1 day in the sample, standard errors were clustered at the student level. Data were analysed using Stata 13. Significance was set at the 10 % level.

## Results

The sample consisted of 968 meals selected by 619 students in grades K-5. About 57 % of students sampled received a free- or reduced-price lunch (Table 1). A majority of students in the sample were White (64 %), 27 % were Black and the remaining 9 % of students were Hispanic or other race/ethnicity. Forty-six per cent of sampled students were female.

**Table 1:** Demographics and milk selection of 619 K-5 students participating in the National School Lunch Program

		Milk choice		
	All students	No milk	Fat-free chocolate	Low-fat white
Receives free- or reduced-price lunch	0·57	0·50	0·58	0·57
	(0·50)	(0·50)	(0·49)	(0·50)
Female	0·46	0·62	0·45	0·38*
	(0·50)	(0·49)	(0·50)	(0·49)
Race				
White	0·64	0·52	0·67	0·58**
	(0·48)	(0·50)	(0·47)	(0·50)
Black	0·27	0·40	0·23	0·35***
	(0·44)	(0·49)	(0·42)	(0·48)
Hispanic	0·04	0·03	0·04	0·03
	(0·19)	(0·18)	(0·19)	(0·16)
Other	0·06	0·05	0·06	0·04
	(0·23)	(0·22)	(0·24)	(0·19)
Number of students	619	58	454	107

*Significant difference between no-milk and low-fat white milk at the 1 % level.

**^,^***Significant difference between fat-free chocolate and low-fat white milk at the 5 % and 1 % level, respectively.

Table 2 presents the energies and nutrients contained in each milk choice offered at the study schools; these data were obtained from the study district’s procurement records and product packaging labels. Both milk options provided were a good source of protein, Ca, Mg, vitamin A and vitamin D. However, the fat-free chocolate milk option included 13 grams of added sugar compared to the low-fat white milk, while the low-fat white milk had one additional gram of saturated fat relative to the fat-free chocolate milk. (Roughly 65 % of total fat found in these milks is saturated fat. We present results for saturated fat instead of total fat because of the 2015–2020 *Dietary Guidelines for Americans* focus on shifting food patterns away from saturated fats and towards unsaturated fats. This recommendation is based on scientific evidence that replacing saturated fat with unsaturated fats is associated with reduced risk of CVD.)

**Table 2:** Energies and nutrients in 8 ounces of low-fat white milk *v*. fat-free chocolate

	Milk flavour		
	Low-fat white	Fat-free chocolate	Percent difference %
Energies (kJ)	102	140	−37
Total sugar (g)	12·69	25·38	−100
Saturated fat (g)	1·55	0·44	72
Protein (g)	8·22	8·55	−4
Ca (mg)	305	288	6
Mg (mg)	27	45	−67
Vitamin A (mcg)	141·82	143	−0·8
Vitamin D (mcg)	2·9	2·8	3

These data were obtained from the study district’s procurement records and product labels.

Table 3 presents the average energies and nutrient content of the meals consumed by the type of milk chosen by the students without controlling for menu offerings or the sociodemographic characteristics of the students (see Appendix B in the supplemental materials for a summary of the nutrient content of meal selections by the type of milk chosen). Approximately 75 % of the selected lunches contained fat-free chocolate milk, 17 % contained low-fat white milk and 8 % contained no milk. Milk consumption rates are statistically different between those choosing the low-fat white milk and those choosing the fat-free chocolate milk; on average, about 70 % (5·6 fluid ounces) of the fat-free chocolate milk and 61 % (4·8 fluid ounces) of low-fat white milk were consumed (*P*-value < 0·01).

**Table 3:** Mean nutrients consumed in National School Lunch Program lunch by type of milk chosen

		Complete lunch			Non-milk meal components		Milk component	
	All lunches	No milk	Low-fat white	Fat-free chocolate	Low-fat white	Fat-free chocolate	Low-fat white	Fat-free chocolate
Prop. milk consumed								
Mean	0·63	–	0·61	0·70				
sd	0·42	–	0·40	0·39				
Energies (kJ)								
Mean	412·85	326·74	377·87	430·08	314·75	331·07	63·12	99·00
sd	157·97	129·50	148·33	158·70	133·65	137·25	42·90	55·80
Total sugar (g)								
Mean	29·13	15·16	22·49	32·15	14·64	14·20	7·85	17·95
sd	16·35	12·05	11·81	16·42	10·59	12·31	5·33	10·12
Saturated fat (g)								
Mean	4·72	4·34	4·92	4·71	3·97	4·40	0·96	0·31
sd	2·39	2·37	2·54	2·36	2·30	2·32	0·65	0·18
Protein (g)								
Mean	18·15	12·42	17·46	18·92	12·37	12·88	5·08	6·05
sd	7·53	5·34	7·55	7·45	5·88	5·97	3·45	3·41
Ca (mg)								
Mean	341·07	160·15	335·76	361·75	147·09	158·16	188·66	203·59
sd	177·42	113·85	187·67	169·49	110·84	116·68	128·09	114·77
Mg (mg)								
Mean	65·00	37·10	54·89	70·30	38·26	38·46	16·64	31·85
sd	31·97	21·35	29·23	31·44	25·59	22·91	11·35	17·93
Vitamin A (mcg)								
Mean	172·71	83·07	160·43	185·15	72·73	84·13	87·70	101·02
sd	146·89	133·24	152·00	143·67	127·19	129·59	59·63	56·99
Vitamin D (mcg)								
Mean	2·08	0·29	2·09	2·27	0·29	0·30	1·80	1·97
sd	1·31	0·38	1·34	1·21	0·37	0·43	1·22	1·11
Number of obs.	968	78	165	725	165	725	165	725

Standard deviation in parentheses.

Students consumed, on average, 413 energies from the complete school lunch. On average, students choosing the fat-free chocolate milk consumed 52 more energies (*P*-value < 0·001) and 10 grams more total sugar (*P*-value < 0·001) than students who chose low-fat white milk with their NSLP lunch. When we separated the complete lunch into milk and non-milk components, we observed that the additional 10 grams of total sugar come from the chocolate milk; total sugar consumed from the non-milk meal components is very similar across milk type chosen with the meal (*P*-value = 0·672). Conversely, the 52 additional energies consumed by students who chose chocolate milk come from both milk and non-milk components: 36 of the additional energies come from milk and 16 energies come from non-milk lunch components. Students who chose chocolate milk also consumed significantly more protein (*P*-value = 0·023), Ca (*P*-value = 0·082), Mg (*P*-value < 0·001) and vitamin A (*P*-value= 0·049) than students who chose low-fat white milk.

Next, we examined the association between socio-economic and demographic characteristics and student’s milk choice using a multinomial logit model. Selected results from the multinomial logit model are shown in Table 4 (results for grade level, school attended and day of the week, and menu controls are presented in Appendix C). The first column contrasts fat-free chocolate milk and low-fat white milk, and the second column compares no milk to low-fat white milk. For completeness, the third column contrasts no milk and fat-free chocolate milk. Black students were statistically less likely than white students to choose fat-free chocolate milk over low-fat white milk (*P*-value = 0·052) and were statistically more likely to select no milk than fat-free chocolate milk (*P*-value = 0·002). Students receiving free- and reduced-price lunch were less likely than those paying full price to choose no milk instead of low-fat white milk (*P*-value = 0·001) or instead of fat-free chocolate milk (*P*-value = 0·064). Female students were more likely than male students to select no milk instead of low-fat white milk (*P*-value = 0·004) or instead of fat-free chocolate milk (*P*-value = 0·014).

**Table 4:** Coefficient estimates from a multinomial logit regression of milk type selection

Outcome	Fat-free chocolate	No milk	No milk
Compared to	Low-fat white	Low-fat white	Fat-free chocolate
Free-/reduced-price lunch			
Mean	0·339	−0·704*	−1·044***
sd	0·27	0·38	0·31
Female			
Mean	0·298	1·057***	0·759**
sd	0·27	0·37	0·31
Race			
Other			
Mean	0·483	0·998	0·515
sd	0·58	0·84	0·65
Black			
Mean	−0·563*	0·482	1·045***
sd	0·29	0·39	0·33
Hispanic			
Mean	−0·17	0·407	0·577
sd	0·76	0·97	0·73
Wald chi-squared	59·82	59·82	59·82

*n* 968. Student-level clustered standard errors are in parentheses.

*^,^**^,^***Significance at the 10 %, 5 % and 1 % level, respectively.

Next, we evaluated how the proportion of milk consumed varied by milk type while controlling for sociodemographic characteristics and menu offerings (school and day of week interactions) (Table 5). We removed the 78 observations where no milk was selected for this analysis. Recall, from the descriptive statistics presented in Table 3, that on average children consume 70 % of their chocolate milk and 61 % of their low-fat white milk without controlling for sociodemographic characteristics. Controlling for sociodemographic characteristics, as shown in Table 5, students who selected fat-free chocolate milk consumed 8·5 % more of their milk compared to students who selected low-fat white milk (*P*-value = 0·039). Students receiving free- or reduced-price lunches consumed 6·2 % more of their milk than students paying full price (*P*-value = 0·082). Black and Hispanic students consumed 14·2 % less of their milk than White students (*P*-value = 0·001). These results suggest milk consumption varies across sociodemographic groups, and hence these groups likely consumed different amounts of key nutrients.

**Table 5:** Regression results for the proportion of milk consumed

	Proportion of milk consumed
Selected fat-free chocolate milk	
Mean	0·0853**
sd	0·0414
Free-/reduced-price lunch	
Mean	0·0617*
sd	0·0355
Female	
Mean	−0·0476
sd	0·034
Race	
Other	
Mean	−0·0894
sd	0·075
Black	
Mean	−0·142***
sd	0·043
Hispanic	
Mean	−0·181*
sd	0·096
Adjusted *R*-squared	0·112

*n* 890. Student-level clustered standard errors are in parentheses.

*^,^**^,^***Significance at the 10 %, 5 % and 1 % level, respectively.

Finally, we used ordinary least square regression analysis to evaluate how milk choice is associated with energies and nutrients consumed from the complete NSLP meal. (The authors also evaluated the association between the milk choice and the selection of key nutrients and total energies. As shown in Appendix F, the results follow similar patterns.) We separated the meal into the non-milk meal components and the milk component to examine whether there is evidence that milk choice is related to the consumption of total energies and nutrients in the non-milk components of the NSLP meal. Each row of Table 6 corresponds to a set of three regressions pertaining to the consumption of the nutrient from the complete meal, from the non-milk meal components and the milk component, respectively. We report only the coefficients describing how low-fat white milk and no milk consumers’ consumption of each nutrient differs relative to those consuming fat-free chocolate milk. We also report the *P*-values for Wald tests of the hypothesis that low-fat white milk consumers and no milk consumers had identical consumptions of each of the nutrients or energies. It is important to recognise that the estimated differences in consumptions between those choosing different types of milk are estimates of partial correlations and not of causal impacts. Sociodemographic and menu offering controls are included as covariates in all regressions and presented in Appendices D, E, and F.

**Table 6:** Regression results, impact of milk choice relative to selecting fat-free chocolate milk on energies and nutrients consumed from the complete lunch, other meal components excluding milk, and milk

	Complete lunch			Non-milk meal components			Milk component		
Dependent variable	Low-fat white	No milk	*P*-value	Low-fat white	No milk	*P*-value	Low-fat white	No milk	*P*-value
Energies (kJ)									
Mean	−58·11***	−114·3***	0·005	−23·19	−19·2	0·825	−34·92***	−95·11***	< 0·001
sd	16·76	14·51		14·47	13·63		5·01	3·71	
Total sugar (g)									
Mean	−9·831***	−17·40***	< 0·001	0·103	−0·153	0·883	−9·934***	−17·25***	< 0·001
sd	1·47	1·67		1·15	1·52		0·75	0·66	
Saturated fat (g)									
Mean	0·208	−0·438*	0·062	−0·441*	−0·142	0·352	0·649***	−0·297***	< 0·001
sd	0·26	0·26		0·24	0·25		0·06	0·02	
Protein (g)									
Mean	−1·538*	−6·696***	< 0·001	−0·638	−0·891	0·750	−0·901**	−5·805***	< 0·001
sd	0·84	0·64		0·64	0·59		0·37	0·23	
Ca (mg)									
Mean	−18·68	−195·1***	< 0·001	−5·899	0·332	0·674	−12·78	−195·4***	< 0·001
sd	20·11	14·57		10·75	11·29		13·38	7·82	
Mg (mg)									
Mean	−17·08***	−33·92***	< 0·001	−2·168	−3·315	0·726	−14·91***	−30·61***	< 0·001
sd	3·21	2·82		2·44	2·46		1·45	1·18	
Vitamin A (mcg)									
Mean	−22·6	−104·4***	< 0·001	−10·32	−7·394	0·874	−12·27*	−96·98***	< 0·001
sd	14·02	17·14		10·98	16·47		6·32	3·86	
Vitamin D (mcg)									
Mean	−0·135	−1·914***	< 0·001	0·0164	−0·0213	0·440	−0·15	−1·892***	< 0·001
sd	0·14	0·09		0·03	0·04		0·13	0·08	

*n* 968. Three regression analyses were performed for each dependent variable to assess the consumption of the dependent variable from the complete lunch and then from the non-milk and milk components of the meal separately. Low-fat white and no milk are the estimated coefficients on the indicator variables representing the student’s choice of milk. *P*-value is *P*-value of the Wald test statistic testing the equivalence of the coefficient on the low-fat white indicator variable with the coefficient on the no milk indicator variable in the same regression. Controls for the student’s sociodemographic characteristics and menu offerings were included as covariates in all regressions. Student-level clustered standard errors are in parentheses.

*^,^**^,^***Significance at the 10 %, 5 % and 1 % level, respectively.

As shown in Table 6, controlling for menu offerings and student sociodemographic characteristics, relative to lunches with fat-free chocolate milk, students choosing low-fat white milk consume 58 fewer energies from their lunches than those selecting fat-free chocolate milk, on average (*P*-value < 0·001). Students who chose low-fat white milk consumed approximately 10 grams, or 40 energies, less total sugar than students selecting fat-free chocolate (*P*-value < 0·001). Compared to students with fat-free chocolate milk, students choosing low-fat white milk consumed 1·5 fewer grams of protein (*P*-value < 0·068) and 17 fewer milligrams of Mg (*P*-value < 0·001) from their complete lunches. These results support the descriptive statistics described above and presented in Table 3; even after controlling for sociodemographic characteristics and menu offerings, students who chose fat-free chocolate milk consume more energies, total sugar, protein and Mg than students who chose low-fat white milk. Relative to students selecting fat-free chocolate milk, students selecting no milk consumed significantly fewer energies and significantly less of all the key nutrients analysed from their NSLP meal. Comparing the consumption of students who selected low-fat white milk to that of the students who selected no milk, the Wald tests indicate that students who selected low-fat white milk consumed significantly more energies, total sugar, saturated fat, protein, Ca, Mg, vitamin A and vitamin D than students who selected no milk.

Repeating the analysis on the non-milk components of the school lunch, only one of the 16 coefficients was statistically significant at the 10 % level. Students who selected low-fat white milk consumed 0·4 fewer grams of saturated fat from the non-milk components of the NSLP than students who selected fat-free chocolate milk (*P*-value = 0·062), after controlling for sociodemographic characteristics and menu offerings.

For completeness, we repeated the analysis on the milk component. Note for observations in which the student did not select a milk, the dependent variables took the value of 0 in these regressions. We found that students selecting fat-free chocolate milk with their meal consumed 35 more energies (*P*-value < 0·001), 10 more grams of total sugar (*P*-value < 0·001), 0·65 fewer grams of saturated fat (*P*-value < 0·001), 0·9 more grams of protein (*P*-value = 0·015), 13 fewer milligrams of Ca (*P*-value = 0·340) and 15 mg more of Mg (*P*-value < 0·001) from the milk component of their meal than students who selected low-fat white milk.

## Discussion

We analysed the sociodemographic determinants of students’ school lunch milk choices. We also examined how the type of milk chosen related to the quantity of milk consumed and the associations between the milk choice and the consumption of energy and nutrients. Our study improves upon prior studies using student-level sociodemographic data instead of school-level controls commonly used in prior studies^(10,11)^.

Given the option between fat-free chocolate milk, low-fat white milk and no milk, the majority of students in our sample selected fat-free chocolate milk. This preference for chocolate milk is similar to the findings of other studies^(2,11,18)^. Going beyond these prior studies, using a multinomial logit, we included student-level sociodemographic controls and found evidence that milk selection varies by gender, race and student’s household’s income (i.e. eligibility to receive free- or reduced-price lunch). For example, Black students were less likely than White students to choose fat-free chocolate milk over low-fat white milk and female students were more likely than male students to select no milk. Furthermore, we found that once selected, the proportion of milk consumed varied not only by flavour but also by the student’s household’s income and race/ethnicity. These findings suggest that recent policy changes reversing the ban on low-fat flavoured milks or potential policy changes such as the elimination of all flavoured milks from schools in New York City or reintroduction of flavoured milks into New Haven schools will have different effects across various sociodemographic groups.

Our novel study combined milk consumption data with nutrient data and examined the consumption of energies and nutrients from the entire meal as well as consumptions from the milk and non-milk meal components separately. Compared to the students who chose low-fat white milk, those who chose fat-free chocolate consumed statistically significantly more energies from their NSLP meal, 60 % of which came from added sugar in chocolate milk. Students who chose fat-free chocolate milk also consumed statistically more Mg and more protein than students who chose low-fat white milk. Students who chose no milk consumed significantly fewer energies and less total sugar, saturated fat, protein, Ca, Mg, vitamin A and vitamin D than students who chose fat-free chocolate milk or low-fat white milk. We intentionally make no claims regarding which consumption pattern is preferable; consumption of additional energies, total sugars and protein might contribute to child overweight and obesity, while consumption of additional micronutrients (Ca, Mg, vitamin A and vitamin D) might be beneficial to the health of students choosing the fat-free chocolate milk. Additional research is needed to determine if the health benefits of consuming these additional micronutrients outweigh the potential increased risk of obesity.

The differences in consumption are driven almost entirely by consumption of the milk component as consumption from the non-milk component was statistically similar across groups with the exception of saturated fat. Overall, the results suggest that the milk component of the meal is an important source of key nutrients and non-milk drinkers miss out on these key nutrients. Furthermore, the findings suggest that if flavoured milk is no longer offered, students currently drinking flavoured milk may decrease their consumption of these key nutrients as Hanks *et al.*^(11)^ found that students drank less milk or selected no milk when flavoured milk was no longer offered. However, we did not find evidence that students who selected chocolate milk consumed more energies from the other non-milk meal components; this may suggest that other than their milk preferences, chocolate milk drinkers have similar food preferences and consumption patterns to white milk drinkers and students who did not drink milk with their lunches. Alternatively, students who selected chocolate milk may have different food preferences and consumption patterns than white milk drinkers, but these differences cannot be detected within this study since we only analyse behaviours at one meal with these two groups selecting from the same limited set of menu offerings.

We found no evidence that students who chose chocolate milk trade off the additional energies in chocolate milk by consuming fewer energies from the other meal components, thus chocolate milk drinkers consumed more energies and total sugar than white milk drinkers and non-milk drinkers. There was some evidence that chocolate milk drinkers traded off the lower levels of saturated fat in the fat-free chocolate milk relative to low-fat white milk by consuming more saturated fat from the other meal components. Students selecting low-fat white milk and fat-free chocolate milk consumed statistically similar amounts of saturated fat from the complete lunch; however, low-fat white milk drinkers consumed significantly more saturated fat from the milk component, while students who selected chocolate milk consumed significantly more saturated fat from the non-milk components. Future studies are needed to examine whether this reflects an actual, important trade-off.

The consumption of both saturated fats and added sugar is particularly important as they are associated with increased cardiovascular risks^(19,20)^. The 2015–2020 *Dietary Guidelines for Americans* encourages food consumption patterns low in saturated fats found naturally in dairy products and recommends drinking skim (fat-free) or low-fat milk instead of reduced fat (2 %) or whole milk^(19)^. The 2015–2020 *Dietary Guidelines* also recommends limiting the consumption of added sugars to less than 10 % of energies per day.

While flavourings increase the likability of milk as indicated by the high rate of chocolate milk selection and higher rates of consumption of chocolate milk than white milk, flavoured milks contain added sugars which detract from their nutritional value. Additional research is needed to examine whether the additional consumption of beneficial nutrients by students resulting from schools offering more acceptable flavoured milk outweighs the increase in added sugar consumptions. Given that the current rates of childhood obesity are higher amongst low-income and minority groups, this paper highlights the need to evaluate the role of unflavoured and flavoured milk within the NSLP as these groups are also more likely to participate in the NSLP^(8)^. Furthermore, flavoured milks are consumed predominantly at school^(21)^. Prior research suggests that children tend to under-consume milk and that the consumption of dairy may decrease the risk of obesity^(19,22,23)^.

The strengths of our study include a large sample size of 968 student-tray observations from 619 students conducted following the implementation of the HHFKA; most prior studies were conducted prior to the HHFKA. While our student population was primarily White with a somewhat limited racial diversity, the population is similar to those studied by Hanks *et al.*^(11)^ and Yon *et al.*^(10)^, so the results can be more readily compared. Further, we examine the relationship between milk consumptions and other nutrients included in the NSLP meal when students were given the choice between flavoured and unflavoured milk as it is the current situation in most elementary schools^(7)^.

Limitations of this study include the examination of milk consumption practices at a single meal. Murphy *et al.*^(18)^ highlight the importance of considering consumption outside of the cafeteria; using 24-h dietary recall data from the National Health and Nutrition Examination Survey (NHANES), they found that the intake of added sugars did not differ between flavoured milk drinkers and non-milk drinkers. Other limitations of the study include the limited time span of the study, and the results only represent a suburban area in a southern state. Furthermore, packed lunches from home were not evaluated. Prior studies indicate that a typical packed lunch contains higher levels of energies, fat, saturated fat and total sugar when compared to NSLP participants’ lunches^(24,25)^. In addition, the schools in our study did not offer other beverages for purchase such as fruit juice or soft drinks and hence might not be generalisable to schools that offer sugar-sweetened beverages. In a study conducted prior to the implementation of the HHFKA, Johnson *et al.*^(26)^ found that children who consumed flavoured milk consumed more Ca but similar percent energy from total fat and added sugars compared with children who did not consume flavoured milk. These authors indicated that the similar levels of added sugar consumption were likely the result of lower intakes of soft drinks and fruit drinks by the children who consumed flavoured milk.

Most importantly, this is an observational correlation study, so we cannot claim causal impacts of milk choice on overall nutrient consumptions. Therefore, we do not know how the results would differ if flavoured milk was no longer offered. Students currently selecting chocolate milk may behave as white milk drinkers if they switched to white milk or as the non-milk drinkers if they stopped selecting milk. Alternatively, they may behave differently or they may instead bring a lunch from home. Schwartz *et al.*^(27)^ found, following the removal of flavoured milk from schools, that student’s consumption of unflavoured milk increased over time; however, the authors did not investigate how this impacted the consumption of meal components. Hanks *et al.*^(11)^ found that 6·8 % fewer students ate the school lunch when chocolate milk was eliminated. Our findings highlight the importance of conducting additional causal analyses of how milk consumption in school can impact children’s nutritional intakes.

## Conclusions

When evaluating the impact of changes in the NSLP offerings, it is prudent to consider that approximately 30 million students participate each day in the NSLP, with an estimated 4·9 billion lunches served each year^(28)^. The NSLP is an opportunity to provide for the nutritional needs of at-risk students as well as to introduce children to healthy foods they could consume over their lifetimes. The changes to the NSLP in HHFKA were enacted to meet this mission. Our study provides evidence that the recent reintroduction of low-fat chocolate milk for students participating in the NSLP should be examined closely for its impacts on future trends in milk consumption patterns, student weight changes over time and the accompanying changes in meal and nutrient consumption patterns.

## Supplementary material

For supplementary material accompanying this paper visit https://doi.org/10.1017/S1368980021000161. click here to view supplementary material
