# Supplementary material for: Students choosing fat-free chocolate milk during school lunch consume more calories, total sugar, protein, minerals and vitamins at lunch
Source: Public Health Nutr. 2021 Jan 14;24(7):1818–27. doi: 10.1017/S1368980021000161 (PMC10195581; doi:10.1017/S1368980021000161)
Supplement: Supplementary file 1 [file S1368980021000161sup001.docx]

Appendix A. Median nutrients chosen in National School Lunch Program lunch by type of milk chosen.

|  |  |  | Complete Lunch | | |  | Non-milk Meal Components | |  | Milk Component | |
| --- | --- | --- | --- | --- | --- | --- | --- | --- | --- | --- | --- |
|  | All Lunches |  | No Milk | Low-fat White | Fat-free Chocolate |  | Low-fat White | Fat-free Chocolate |  | Low-fat White | Fat-free Chocolate |
| Prop. Milk Consumed | 0.88 |  |  | 0.70 | 1.00 |  |  |  |  |  |  |
|  | [0.85] |  |  | [0.80] | [0.68] |  |  |  |  |  |  |
| Calories (kcal) | 413.00 ^a^ |  | 327.00^a^ | 387.33 | 432.00 ^a^ |  | 319.00 | 332.00 |  | 74.00 | 140.00 |
|  | [190.33] |  | [161.00] | [171.00] | [204.00] |  | [150.00] | [171.00] |  | [82.00] | [95.67] |
| Total Sugar (g) | 28.11 |  | 12.88 | 20.37 | 29.91 |  | 12.94 | 11.14 |  | 9.14 | 25.38 |
|  | [22.47] |  | [18.10] | [16.34] | [21.48] |  | [17.85] | [18.04] |  | [10.15] | [17.35] |
| Saturated Fat (g) | 5.09 |  | 4.96 | 4.86 | 5.15 |  | 3.89 | 4.79 |  | 1.11 | 0.44 |
|  | [3.39] |  | [3.40] | [3.75] | [3.31] |  | [3.23] | [3.20] |  | [1.24] | [0.30] |
| Protein (g) | 18.30 |  | 12.31^a^ | 17.77 ^a^ | 19.25 |  | 12.72 | 13.39 |  | 5.92 | 8.55 |
|  | [9.87] |  | [5.57] | [9.02] | [9.28] |  | [6.00] | [6.42] |  | [6.58] | [5.84] |
| Calcium (mg) | 345.17 |  | 160.17 | 350.00 | 360.00 |  | 133.00 | 162.00 |  | 8.77 | 27.35 |
|  | [259.17] |  | [175.00] | [253.33] | [227.00] |  | [152.00] | [170.00] |  | [9.74] | [18.69] |
| Magnesium (mg) | 65.00 |  | 33.00 | 50.00 | 69.67 |  | 30.67 | 33.00 |  | 19.00 | 45.00 |
|  | [42.50] |  | [18.67] | [34.00] | [42.00] |  | [23.00] | [27.00] |  | [22.00] | [31.00] |
| Vitamin A (mcg) | 157.00 |  | 49.50 | 151.00 | 166.00 |  | 45.00 | 51.67 |  | 102.00 | 143.00 |
|  | [123.00] |  | [70.00] | [117.00] | [99.00] |  | [64.00] | [67.00] |  | [114.00] | [97.67] |
| Vitamin D (mcg) | 2.68 |  | 0.20 | 2.40 | 2.80 |  | 0.20 | 0.10 |  | 2.10 | 2.80 |
|  | [2.25] |  | [0.20] | [2.30] | [1.67] |  | [0.20] | [0.20] |  | [2.30] | [1.93] |
| Number of Obs. | 968 |  | 78 | 165 | 725 |  | 165 | 725 |  | 165 | 725 |

Notes: Interquartile Range in brackets below the median. Nutrients were tested for normality using the Shapiro–Wilk normality test. The superscript ^a^ represents a Shapiro-Wilk p-value greater than 0.05.

Appendix B. Mean nutrients selected in National School Lunch Program lunch by type of milk chosen.

|  |  |  | Complete Lunch | | |  | Non-milk Meal Components | |  | Milk Component | |
| --- | --- | --- | --- | --- | --- | --- | --- | --- | --- | --- | --- |
|  | All Lunches |  | No Milk | Low-fat White | Fat-free Chocolate |  | Low-fat White | Fat-free Chocolate |  | Low-fat White | Fat-free Chocolate |
| Calories (kcal) | 603.47 |  | 475.81 | 579.34 | 622.69 |  | 475.47 | 481.72 |  | 103.87 | 140.97 |
|  | (116.95) |  | (100.84) | (119.13) | (108.22) |  | (117.82) | (107.42) |  | (13.80) | (11.59) |
| Total Sugar (g) | 40.83 |  | 23.53 | 33.32 | 44.40 |  | 22.17 | 21.30 |  | 11.15 | 23.10 |
|  | (16.31) |  | (15.28) | (12.93) | (15.32) |  | (12.16) | (13.24) |  | (4.81) | (7.84) |
| Saturated Fat (g) | 5.86 |  | 5.27 | 6.50 | 5.78 |  | 5.14 | 5.38 |  | 1.36 | 0.40 |
|  | (2.40) |  | (2.31) | (2.53) | (2.35) |  | (2.46) | (2.33) |  | (0.59) | (0.14) |
| Protein (g) | 23.31 |  | 16.00 | 23.43 | 24.07 |  | 16.21 | 16.28 |  | 7.22 | 7.79 |
|  | (7.01) |  | (4.99) | (6.96) | (6.76) |  | (6.27) | (5.88) |  | (3.12) | (2.65) |
| Calcium (mg) | 441.96 |  | 206.33 | 462.63 | 462.60 |  | 194.51 | 200.42 |  | 268.12 | 262.18 |
|  | (175.04) |  | (136.02) | (176.92) | (159.25) |  | (122.17) | (127.76) |  | (115.69) | (89.03) |
| Magnesium (mg) | 88.25 |  | 54.29 | 80.75 | 93.61 |  | 57.07 | 52.98 |  | 23.68 | 40.63 |
|  | (35.01) |  | (26.49) | (37.80) | (32.76) |  | (35.22) | (28.24) |  | (10.79) | (14.47) |
| Vitamin A (mcg) | 266.76 |  | 125.62 | 252.98 | 285.08 |  | 128.62 | 155.53 |  | 124.36 | 129.55 |
|  | (215.12) |  | (187.20) | (210.08) | (213.45) |  | (201.92) | (210.66) |  | (53.79) | (44.19) |
| Vitamin D (mcg) | 2.68 |  | 0.37 | 2.92 | 2.87 |  | 0.35 | 0.37 |  | 2.56 | 2.51 |
|  | (1.25) |  | (0.48) | (1.24) | (1.04) |  | (0.44) | (0.50) |  | (1.16) | (0.90) |
| Number of Obs. | 968 |  | 78 | 165 | 725 |  | 165 | 725 |  | 165 | 725 |

Notes: Standard deviation in parentheses.

Appendix C. Complete results from a multinomial logit regression of milk type selection

| Outcome: | Fat-free Chocolate | No Milk | No Milk |
| --- | --- | --- | --- |
| *Compared to:* | *Low-fat White* | *Low-fat White* | *Fat-free Chocolate* |
| Receives Free or Reduced-Price Lunch | 0.339 | -0.704* | -1.044*** |
|  | (0.274) | (0.375) | (0.314) |
| Female | 0.298 | 1.057*** | 0.759** |
|  | (0.265) | (0.370) | (0.313) |
| Race |  |  |  |
| Other | 0.483 | 0.998 | 0.515 |
|  | (0.579) | (0.837) | (0.653) |
| Black | -0.563* | 0.482 | 1.045*** |
|  | (0.294) | (0.387) | (0.330) |
| Hispanic | -0.17 | 0.407 | 0.577 |
|  | (0.758) | (0.967) | (0.728) |
| Grade Level |  |  |  |
| First | -0.567 | 0.687 | 1.254* |
|  | (0.451) | (0.759) | (0.646) |
| Second | -0.645 | 1.092 | 1.737** |
|  | (0.476) | (0.782) | (0.678) |
| Third | -0.826** | 0.916 | 1.742*** |
|  | (0.412) | (0.720) | (0.621) |
| Fourth | -0.397 | 0.718 | 1.115* |
|  | (0.422) | (0.760) | (0.661) |
| Fifth | -0.23 | 1.502** | 1.733*** |
|  | (0.490) | (0.756) | (0.622) |
| Day of the Week |  |  |  |
| Tuesday | 1.252*** | 1.264** | 0.0118 |
|  | (0.365) | (0.569) | (0.519) |
| Wednesday | 0.577* | 0.522 | -0.0553 |
|  | (0.336) | (0.531) | (0.470) |
| Thursday | 0.755** | 0.675 | -0.0805 |
|  | (0.373) | (0.557) | (0.481) |
| Friday | 0.617** | 0.75 | 0.133 |
|  | (0.303) | (0.503) | (0.460) |
| School |  |  |  |
| School 2 | 0.295 | 0.191 | -0.104 |
|  | (0.354) | (0.590) | (0.542) |
| School-Day Interactions |  |  |  |
| School 1 x Day 2 | -1.127** | -1.174 | -0.0467 |
|  | (0.460) | (0.832) | (0.767) |
| School 1 x Day 3 | -0.63 | -0.125 | 0.505 |
|  | (0.428) | (0.743) | (0.673) |
| School 1 x Day 4 | -0.662 | -0.407 | 0.255 |
|  | (0.478) | (0.759) | (0.670) |
| School 1 x Day 5 | -0.479 | -1.154 | -0.674 |
|  | (0.426) | (0.851) | (0.788) |
| Constant | 1.281*** | -2.561*** | -3.843*** |
|  | (0.492) | (0.765) | (0.652) |
| Observations | 968 | 968 | 968 |

Notes: N=968. Student-level clustered standard errors are in parentheses *, **, ***, represent significance at the 10%, 5%, and 1% level, respectively.

Appendix D. Complete regression results, impact of milk choice relative to selecting fat-free chocolate milk on calories and nutrients consumed from complete lunch

|  | Consumed from Complete Lunch | | | | | | | |
| --- | --- | --- | --- | --- | --- | --- | --- | --- |
|  | Calories | Total Sugar | Saturated Fat | Protein | Calcium | Magnesium | Vit A | Vit D |
| Milk Choice |  |  |  |  |  |  |  |  |
| No Milk | -114.3*** | -17.40*** | -0.438* | -6.696*** | -195.1*** | -33.92*** | -104.4*** | -1.914*** |
|  | (14.51) | (1.67) | (0.26) | (0.64) | (14.57) | (2.82) | (17.14) | (0.09) |
| 1% Milk | -58.11*** | -9.831*** | 0.208 | -1.538* | -18.68 | -17.08*** | -22.6 | -0.135 |
|  | (16.76) | (1.47) | (0.26) | (0.84) | (20.11) | (3.21) | (14.02) | (0.14) |
| Receives Free or Reduced-Price Lunch | 11.26 | 2.112 | 0.134 | 0.628 | 12.23 | 2.373 | 13.04 | 0.14 |
|  | (12.58) | (1.30) | (0.17) | (0.60) | (13.04) | (2.50) | (9.67) | (0.10) |
| Female | -19.56 | -1.917 | -0.0682 | -0.974* | -19.28 | -4.239* | 9.268 | -0.147 |
|  | (11.97) | (1.23) | (0.16) | (0.57) | (12.72) | (2.39) | (9.85) | (0.10) |
| Race |  |  |  |  |  |  |  |  |
| Other | -19.56 | -2.529 | 0.0209 | -0.764 | -19.12 | -3.215 | -14.66 | -0.192 |
|  | (21.47) | (3.12) | (0.37) | (1.09) | (30.00) | (4.74) | (25.17) | (0.21) |
| Black | -19.01 | -1.622 | -0.206 | -1.624** | -43.13*** | -6.902** | -31.56*** | -0.358*** |
|  | (14.67) | (1.51) | (0.20) | (0.67) | (16.17) | (2.80) | (11.04) | (0.12) |
| Hispanic | -8.991 | -6.253** | 0.394 | 0.0502 | -52.53 | -7.645 | -28.41 | -0.518* |
|  | (33.20) | (2.81) | (0.51) | (1.76) | (32.29) | (7.12) | (30.13) | (0.27) |
| Grade Level |  |  |  |  |  |  |  |  |
| First | 47.99** | 1.208 | 0.544** | 1.362 | 11.91 | 4.557 | 17.71 | -0.144 |
|  | (18.75) | (2.01) | (0.27) | (0.91) | (22.29) | (3.58) | (13.66) | (0.18) |
| Second | 71.43*** | 2.718 | 0.747** | 1.769* | 24.8 | 8.969** | 45.02** | -0.0653 |
|  | (21.77) | (2.24) | (0.29) | (0.97) | (23.17) | (4.20) | (17.44) | (0.18) |
| Third | 104.2*** | 4.893** | 1.313*** | 3.873*** | 54.86*** | 16.31*** | 49.51*** | 0.303* |
|  | (20.26) | (2.11) | (0.30) | (0.92) | (21.21) | (3.94) | (15.66) | (0.17) |
| Fourth | 99.23*** | 3.677** | 1.136*** | 4.812*** | 51.79*** | 16.53*** | 42.28*** | 0.294* |
|  | (17.21) | (1.86) | (0.25) | (0.85) | (18.43) | (3.35) | (13.09) | (0.15) |
| Fifth | 119.3*** | 4.900** | 1.296*** | 5.115*** | 61.65*** | 17.60*** | 38.93** | 0.262* |
|  | (17.63) | (2.08) | (0.26) | (0.86) | (19.44) | (3.53) | (15.50) | (0.16) |
| Day of the Week |  |  |  |  |  |  |  |  |
| Tuesday | -119.3*** | -6.946*** | -1.299*** | -2.377** | 1.497 | -26.09*** | -22.93 | 0.248* |
|  | (19.00) | (1.95) | (0.27) | (0.94) | (23.32) | (4.20) | (28.67) | (0.15) |
| Wednesday | -9.071 | -13.65*** | -0.804** | 0.468 | -142.9*** | -20.57*** | 22.3 | 0.182 |
|  | (19.36) | (1.67) | (0.35) | (0.81) | (17.24) | (4.03) | (34.79) | (0.14) |
| Thursday | -102.9*** | -16.69*** | -1.199*** | -4.086*** | -98.57*** | -34.87*** | -115.6*** | -0.591*** |
|  | (18.82) | (1.76) | (0.27) | (0.87) | (17.89) | (4.16) | (25.20) | (0.14) |
| Friday | -63.56*** | -1.562 | -1.347*** | -2.808*** | -42.31** | -11.35** | -71.97*** | -0.344*** |
|  | (18.99) | (1.90) | (0.27) | (0.85) | (18.12) | (4.58) | (25.86) | (0.13) |
| School |  |  |  |  |  |  |  |  |
| School 2 | -127.8*** | -16.60*** | -3.807*** | -3.104*** | -209.8*** | -11.08** | -124.5*** | -0.814*** |
|  | (21.98) | (1.84) | (0.27) | (1.04) | (19.34) | (5.26) | (27.67) | (0.14) |
| School-Day Interactions |  |  |  |  |  |  |  |  |
| School 1 x Day 2 | 105.1*** | 8.710*** | 3.443*** | 2.138 | 93.02*** | -0.793 | 58.09* | -0.123 |
|  | (26.17) | (2.66) | (0.36) | (1.41) | (29.19) | (5.77) | (31.99) | (0.20) |
| School 1 x Day 3 | -40.16 | 11.48*** | 1.509*** | -1.993 | 121.5*** | -1.049 | -24.79 | 0.0526 |
|  | (26.03) | (2.36) | (0.41) | (1.25) | (24.03) | (5.59) | (36.73) | (0.19) |
| School 1 x Day 4 | 51.88** | 18.79*** | 3.772*** | 5.041*** | 233.7*** | 13.72** | 154.1*** | 1.285*** |
|  | (25.82) | (2.47) | (0.39) | (1.28) | (26.34) | (5.81) | (28.63) | (0.20) |
| School 1 x Day 5 | 17.9 | -0.133 | 3.979*** | -0.786 | 193.6*** | -6.794 | 100.1*** | 0.790*** |
|  | (26.18) | (2.58) | (0.38) | (1.21) | (26.29) | (6.11) | (28.61) | (0.20) |
| Constant | 478.0*** | 42.18*** | 5.459*** | 19.65*** | 441.8*** | 86.93*** | 222.8*** | 2.590*** |
|  | (20.59) | (1.89) | (0.33) | (0.97) | (20.66) | (4.39) | (24.50) | (0.16) |
| R-squared | 0.282 | 0.291 | 0.242 | 0.205 | 0.308 | 0.299 | 0.177 | 0.283 |

Notes: N=968. Student-level clustered standard errors are in parentheses. *, **, ***, represent significance at the 10%, 5%, and 1% level, respectively.

Appendix E. Complete regression results, impact of milk choice relative to selecting fat-free chocolate milk on calories and nutrients consumed from other meal components excluding milk

|  | Consumed from other meal components excluding milk | | | | | | | |
| --- | --- | --- | --- | --- | --- | --- | --- | --- |
|  | Calories | Total Sugar | Saturated Fat | Protein | Calcium | Magnesium | Vit A | Vit D |
| Milk Choice |  |  |  |  |  |  |  |  |
| No Milk | -19.2 | -0.153 | -0.142 | -0.891 | 0.332 | -3.315 | -7.394 | -0.0213 |
|  | (13.63) | (1.52) | (0.25) | (0.59) | (11.29) | (2.46) | (16.47) | (0.04) |
| 1% Milk | -23.19 | 0.103 | -0.441* | -0.638 | -5.899 | -2.168 | -10.32 | 0.0164 |
|  | (14.47) | (1.15) | (0.24) | (0.64) | (10.75) | (2.44) | (10.98) | (0.03) |
| Receives Free or Reduced-Price Lunch | 4.958 | 0.972 | 0.114 | 0.241 | -0.824 | 0.363 | 6.536 | 0.0122 |
|  | (10.56) | (0.94) | (0.16) | (0.47) | (7.27) | (1.71) | (7.93) | (0.02) |
| Female | -12.51 | -0.598 | -0.0545 | -0.557 | -5.424 | -1.919 | 16.23** | -0.0103 |
|  | (10.04) | (0.91) | (0.15) | (0.44) | (7.00) | (1.66) | (8.16) | (0.02) |
| Race |  |  |  |  |  |  |  |  |
| Other | -7.333 | -0.212 | 0.0369 | -0.0554 | 4.235 | 0.785 | -2.914 | 0.0378 |
|  | (16.68) | (2.26) | (0.37) | (0.75) | (18.11) | (2.84) | (21.28) | (0.05) |
| Black | -2.1 | 1.309 | -0.125 | -0.546 | -6.167 | -1.608 | -13.42 | -0.000533 |
|  | (12.33) | (1.09) | (0.19) | (0.52) | (8.71) | (1.85) | (8.65) | (0.03) |
| Hispanic | 14.79 | -1.93 | 0.467 | 1.501 | -3.7 | -0.0244 | -4.169 | -0.0387 |
|  | (25.50) | (1.71) | (0.47) | (1.20) | (13.83) | (4.42) | (23.74) | (0.05) |
| Grade Level |  |  |  |  |  |  |  |  |
| First | 55.95*** | 2.613* | 0.577** | 1.861** | 28.93** | 7.095*** | 26.08*** | 0.0192 |
|  | (16.04) | (1.43) | (0.26) | (0.74) | (11.55) | (2.34) | (10.05) | (0.04) |
| Second | 76.76*** | 3.617** | 0.779*** | 2.120*** | 36.95*** | 10.63*** | 50.92*** | 0.046 |
|  | (18.61) | (1.66) | (0.28) | (0.78) | (12.45) | (2.76) | (14.15) | (0.04) |
| Third | 94.52*** | 3.309** | 1.249*** | 3.228*** | 32.31*** | 13.38*** | 38.57*** | 0.0811* |
|  | (17.42) | (1.51) | (0.29) | (0.76) | (12.37) | (2.65) | (12.42) | (0.04) |
| Fourth | 87.91*** | 1.657 | 1.094*** | 4.111*** | 28.02** | 12.93*** | 30.50*** | 0.0609 |
|  | (15.10) | (1.41) | (0.25) | (0.72) | (11.95) | (2.16) | (10.92) | (0.04) |
| Fifth | 110.6*** | 3.321** | 1.272*** | 4.591*** | 44.04*** | 14.80*** | 30.13** | 0.0863** |
|  | (15.07) | (1.58) | (0.26) | (0.70) | (12.43) | (2.20) | (12.99) | (0.04) |
| Day of the Week |  |  |  |  |  |  |  |  |
| Tuesday | -104.8*** | -4.464** | -1.223*** | -1.441* | 33.71* | -21.59*** | -7.183 | 0.560*** |
|  | (17.32) | (1.74) | (0.27) | (0.78) | (19.07) | (3.59) | (27.61) | (0.07) |
| Wednesday | -6.704 | -13.21*** | -0.798** | 0.611 | -138.2*** | -19.80*** | 24.67 | 0.232*** |
|  | (18.35) | (1.29) | (0.36) | (0.71) | (10.90) | (3.54) | (34.34) | (0.06) |
| Thursday | -84.26*** | -13.54*** | -1.094*** | -2.875*** | -56.71*** | -29.11*** | -95.16*** | -0.181*** |
|  | (16.53) | (1.24) | (0.26) | (0.69) | (10.08) | (3.44) | (24.16) | (0.03) |
| Friday | -58.07*** | -0.793 | -1.284*** | -2.400*** | -27.51** | -9.816** | -65.00*** | -0.201*** |
|  | (17.42) | (1.62) | (0.26) | (0.71) | (10.93) | (4.13) | (25.00) | (0.03) |
| School |  |  |  |  |  |  |  |  |
| School 2 | -99.16*** | -11.82*** | -3.629*** | -1.212 | -144.1*** | -2.287 | -92.59*** | -0.179*** |
|  | (19.74) | (1.44) | (0.26) | (0.86) | (11.99) | (4.47) | (26.32) | (0.03) |
| School-Day Interactions |  |  |  |  |  |  |  |  |
| School 1 x Day 2 | 87.94*** | 5.801*** | 3.345*** | 1.016 | 54.30** | -6.137 | 39.22 | -0.496*** |
|  | (23.74) | (2.19) | (0.36) | (1.20) | (22.31) | (4.65) | (30.52) | (0.07) |
| School 1 x Day 3 | -50.37** | 9.718*** | 1.458*** | -2.649** | 99.02*** | -4.266 | -35.78 | -0.166** |
|  | (23.72) | (1.75) | (0.41) | (1.06) | (14.69) | (4.60) | (35.69) | (0.07) |
| School 1 x Day 4 | 7.024 | 11.36*** | 3.484*** | 2.062** | 130.0*** | -0.035 | 103.8*** | 0.281*** |
|  | (22.33) | (1.76) | (0.38) | (1.03) | (16.16) | (4.55) | (26.97) | (0.04) |
| School 1 x Day 5 | 1.117 | -2.785 | 3.846*** | -1.939** | 153.0*** | -11.84** | 80.65*** | 0.400*** |
|  | (23.40) | (2.00) | (0.37) | (0.97) | (16.01) | (5.21) | (27.02) | (0.05) |
| Constant | 361.9*** | 21.33*** | 5.056*** | 12.50*** | 200.1*** | 49.79*** | 103.2*** | 0.251*** |
|  | (19.49) | (1.57) | (0.32) | (0.86) | (14.31) | (3.73) | (22.85) | (0.04) |
| R-squared | 0.266 | 0.231 | 0.235 | 0.158 | 0.341 | 0.28 | 0.128 | 0.308 |

Notes: N=968. Student-level clustered standard errors are in parentheses. *, **, ***, represent significance at the 10%, 5%, and 1% level, respectively.

Appendix F. Complete regression results, impact of milk choice relative to selecting fat-free chocolate milk on calories and nutrients consumed from milk

|  | Consumed from milk | | | | | | | |
| --- | --- | --- | --- | --- | --- | --- | --- | --- |
|  | Calories | Total Sugar | Saturated Fat | Protein | Calcium | Magnesium | Vit A | Vit D |
| Milk Choice |  |  |  |  |  |  |  |  |
| No Milk | -95.11*** | -17.25*** | -0.297*** | -5.805*** | -195.4*** | -30.61*** | -96.98*** | -1. 892*** |
|  | (3.71) | (0.66) | (0.02) | (0.23) | (7.82) | (1.18) | (3.86) | (0.08) |
| 1% Milk | -34.92*** | -9.934*** | 0.649*** | -0.901** | -12.78 | -14.91*** | -12.27* | -0.152 |
|  | (5.01) | (0.75) | (0.06) | (0.37) | (13.38) | (1.45) | (6.32) | (0.13) |
| Receives Free or Reduced-Price Lunch | 6.307 | 1.14 | 0.0208 | 0.387 | 13.05 | 2.01 | 6.501 | 0.128 |
|  | (4.50) | (0.79) | (0.03) | (0.29) | (9.81) | (1.42) | (4.81) | (0.09) |
| Female | -7.053 | -1.319* | -0.0137 | -0.417 | -13.86 | -2.320* | -6.964 | -0.137 |
|  | (4.36) | (0.77) | (0.03) | (0.28) | (9.51) | (1.37) | (4.66) | (0.09) |
| Race |  |  |  |  |  |  |  |  |
| Other | -12.23 | -2.317 | -0.016 | -0.709 | -23.36 | -4 | -11.75 | -0.23 |
|  | (9.63) | (1.72) | (0.04) | (0.60) | (20.36) | (3.06) | (10.06) | (0.20) |
| Black | -16.91*** | -2.930*** | -0.0814*** | -1.078*** | -36.97*** | -5.294*** | -18.14*** | -0.358*** |
|  | (5.43) | (0.96) | (0.03) | (0.34) | (11.74) | (1.72) | (5.76) | (0.11) |
| Hispanic | -23.78** | -4.323** | -0.073 | -1.451* | -48.83* | -7.621** | -24.24* | -0.479* |
|  | (11.84) | (2.07) | (0.07) | (0.76) | (26.08) | (3.71) | (12.77) | (0.25) |
| Grade Level |  |  |  |  |  |  |  |  |
| First | -7.953 | -1.405 | -0.033 | -0.499 | -17.02 | -2.538 | -8.364 | -0.163 |
|  | (8.13) | (1.45) | (0.04) | (0.51) | (17.36) | (2.59) | (8.55) | (0.17) |
| Second | -5.331 | -0.899 | -0.0318 | -0.351 | -12.15 | -1.659 | -5.9 | -0.111 |
|  | (8.14) | (1.43) | (0.05) | (0.52) | (17.68) | (2.57) | (8.67) | (0.17) |
| Third | 9.639 | 1.583 | 0.0649* | 0.644 | 22.54 | 2.931 | 10.94 | 0.222 |
|  | (7.59) | (1.35) | (0.04) | (0.48) | (16.20) | (2.41) | (7.98) | (0.16) |
| Fourth | 11.33 | 2.021 | 0.0421 | 0.701 | 23.76 | 3.598 | 11.78 | 0.233 |
|  | (7.27) | (1.29) | (0.04) | (0.46) | (15.49) | (2.31) | (7.63) | (0.15) |
| Fifth | 8.655 | 1.579 | 0.0248 | 0.524 | 17.61 | 2.806 | 8.805 | 0.176 |
|  | (7.20) | (1.28) | (0.04) | (0.45) | (15.44) | (2.29) | (7.60) | (0.15) |
| Day of the Week |  |  |  |  |  |  |  |  |
| Tuesday | -14.50** | -2.482** | -0.0764** | -0.936** | -32.21** | -4.500** | -15.75** | -0.312** |
|  | (6.18) | (1.09) | (0.04) | (0.39) | (13.39) | (1.95) | (6.58) | (0.13) |
| Wednesday | -2.368 | -0.437 | -0.00576 | -0.142 | -4.746 | -0.763 | -2.373 | -0.0501 |
|  | (5.94) | (1.04) | (0.04) | (0.38) | (13.04) | (1.87) | (6.39) | (0.13) |
| Thursday | -18.60*** | -3.150*** | -0.105*** | -1.212*** | -41.87*** | -5.768*** | -20.44*** | -0.410*** |
|  | (6.48) | (1.13) | (0.04) | (0.41) | (14.25) | (2.04) | (6.98) | (0.14) |
| Friday | -5.485 | -0.769 | -0.0632* | -0.408 | -14.8 | -1.535 | -6.975 | -0.144 |
|  | (5.58) | (0.97) | (0.04) | (0.36) | (12.47) | (1.75) | (6.09) | (0.12) |
| School |  |  |  |  |  |  |  |  |
| School 2 | -28.64*** | -4.773*** | -0.178*** | -1.891*** | -65.69*** | -8.797*** | -31.90*** | -0.635*** |
|  | (6.46) | (1.12) | (0.05) | (0.42) | (14.55) | (2.02) | (7.09) | (0.14) |
| School-Day Interactions |  |  |  |  |  |  |  |  |
| School 1 x Day 2 | 17.20* | 2.909* | 0.0978* | 1.121* | 38.72* | 5.344* | 18.87* | 0.373* |
|  | (9.09) | (1.59) | (0.06) | (0.58) | (20.04) | (2.86) | (9.80) | (0.19) |
| School 1 x Day 3 | 10.2 | 1.759 | 0.0519 | 0.656 | 22.52 | 3.217 | 10.99 | 0.219 |
|  | (8.50) | (1.49) | (0.05) | (0.55) | (18.77) | (2.67) | (9.18) | (0.18) |
| School 1 x Day 4 | 44.85*** | 7.427*** | 0.289*** | 2.979*** | 103.7*** | 13.76*** | 50.30*** | 1.004*** |
|  | (9.38) | (1.64) | (0.06) | (0.60) | (20.64) | (2.95) | (10.10) | (0.20) |
| School 1 x Day 5 | 16.78* | 2.652* | 0.133** | 1.153** | 40.67** | 5.043* | 19.49** | 0.390** |
|  | (8.79) | (1.53) | (0.06) | (0.56) | (19.44) | (2.76) | (9.50) | (0.19) |
| Constant | 116.1*** | 20.85*** | 0.404*** | 7.151*** | 241.7*** | 37.15*** | 119.6*** | 2.340*** |
|  | (7.22) | (1.27) | (0.04) | (0.46) | (15.82) | (2.28) | (7.75) | (0.15) |
| R-squared | 0.304 | 0.353 | 0.47 | 0.273 | 0.267 | 0.33 | 0.271 | 0.267 |

Notes: N=968. Student-level clustered standard errors are in parentheses. *, **, ***, represent significance at the 10%, 5%, and 1% level, respectively.

Appendix G. Regression results, impact of milk choice relative to selecting fat-free chocolate milk on calories and nutrients *selected* from the complete lunch, other meal components excluding milk, and milk

|  |  | Complete Lunch | | |  | Non-Milk Meal Components | | |  | Milk Component | | |
| --- | --- | --- | --- | --- | --- | --- | --- | --- | --- | --- | --- | --- |
|  |  | Low-fat White | No Milk | p-value |  | Low-fat White | No Milk | p-value |  | Low-fat White | No Milk | p-value |
| Dependent Variable |  |  |  |  |  |  |  |  |  |  |  |  |
| Calories (kcal) |  | -44.31*** | -149.7*** | <0.001 |  | -6.949 | -8.426 | 0.903 |  | -37.36*** | -141.2*** | <0.001 |
|  |  | (8.08) | (10.41) |  |  | (7.78) | (10.35) |  |  | (1.40) | (0.54) |  |
| Total Sugar (g) |  | -10.82*** | -21.27*** | <0.001 |  | 0.855 | 1.306 | 0.824 |  | -11.67*** | -22.58*** | <0.001 |
|  |  | (1.35) | (1.90) |  |  | (1.17) | (1.82) |  |  | (0.60) | (0.53) |  |
| Saturated Fat (g) |  | 0.809*** | -0.464** | <0.001 |  | -0.154 | -0.0745 | 0.769 |  | 0.964*** | -0.389*** | <0.001 |
|  |  | (0.21) | (0.23) |  |  | (0.20) | (0.23) |  |  | (0.05) | (0.01) |  |
| Protein (g) |  | -0.456 | -8.069*** | <0.001 |  | 0.0105 | -0.463 | 0.502 |  | -0.47 | -7.607*** | <0.001 |
|  |  | (0.65) | (0.58) |  |  | (0.55) | (0.56) |  |  | (0.31) | (0.18) |  |
| Calcium (mg) |  | 13.39 | -246.5*** | <0.001 |  | 3.98 | 9.564 | 0.704 |  | 9.41 | -256.0*** | <0.001 |
|  |  | (16.45) | (14.32) |  |  | (9.63) | (12.25) |  |  | (11.41) | (6.18) |  |
| Magnesium (mg) |  | -14.90*** | -39.98*** | <0.001 |  | 1.726 | -0.154 | 0.588 |  | -16.62*** | -39.83*** | <0.001 |
|  |  | (3.05) | (2.83) |  |  | (2.63) | (2.67) |  |  | (1.19) | (0.95) |  |
| Vitamin A (mcg) |  | -30.63 | -168.0*** | <0.001 |  | -26.98 | -41.41* | 0.606 |  | -3.64 | -126.6*** | <0.001 |
|  |  | (20.89) | (22.89) |  |  | (19.71) | (22.78) |  |  | (5.35) | (3.05) |  |
| Vitamin D (mcg) |  | 0.103 | -2.464*** | <0.001 |  | 0.0256 | -0.00513 | 0.586 |  | 0.08 | -2.459*** | <0.001 |
|  |  | (0.12) | (0.08) |  |  | (0.04) | (0.05) |  |  | (0.11) | (0.06) |  |

Notes: N=968. Three regression analyses were performed for each dependent variable to assess the selection of the dependent variable from the complete lunch and then from the non-milk and milk components of the meal separately. Low-fat white and no milk are the estimated coefficients on the indicator variables representing the student’s choice of milk. P-value is p-value of the Wald test statistic testing the equivalence of the coefficient on the low-fat white indicator variable with the coefficient on the no milk indicator variable in the same regression. Controls for the student’s sociodemographic characteristics and menu offerings were included as covariates in all regressions. Student-level clustered standard errors are in parentheses. *, **, ***, represent significance at the 10%, 5%, and 1% level, respectively.
